# Valedictory Address to the Graduating Class on Behalf of the Faculty of Atlanta Medical College, at the Annual Commencement, August 31st, 1866

**Published:** 1866-10

**Authors:** Henry Jackson


### ARTICLE III.

*Valedictory Address to the Graduating Class on behalf of the Faculty of Atlanta Medical College, at the Annual Commencement, August 31st, 1866.* By Henry Jackson, Esq.

Gentlemen of the Graduating Class:

By request of the Faculty of this Institution, the duty devolves upon me this morning of pronouncing to you their final farewell; of severing the last link of the chain that has hitherto bound you and them together; you as students, them as professors; you as earnest seekers after knowledge, them as ever-gushing fountains of wisdom, from which you have drank the pure and sparkling waters. This is a duty both painful and pleasant: painful in rending asunder the close connection of friendship and love which must ever exist between those who tread together the rough and tedious paths of learning; pleasant in anticipation of the brilliant future that awaits you and the honor with which your success will crown not only yourselves but them also.

With nerves and sinews disciplined for the conflict, you are embarking upon the great Ocean of Life; to be tossed by its billows, to be driven by its blasts, to be dazzled by its sunshine. With chart in hand you commence your voyage: whether its closing scenes shall find you laden with honors, which joyous anticipation holds temptingly forth, or whether, as stranded wrecks, you will sink into oblivion—your past existence marked alone by the scattered fragments of hope's young argosy, and the mourning and seething of the engulfing waters—is a question to be solved by the future. The test has yet to be applied: do but your duty, and the solution will be pleasant.

You have embraced a profession which is justly styled learned, and yours will be the part to sustain its title. We live in a progressive age: the revolving years pour forth countless innovations upon science, and what the human mind of yesterday could but conceive of, becomes the common place of to-day. All the energy, all the perseverance of the youth of the present generation will be required to keep pace with the rapid strides of improvement. To keep pace, did I say? This is not sufficient: it will be theirs to lead. You have passed through a great revolution; you have seen old principles overturned and new ones established; you have followed the hearse of the short-lived but glorious Southern Confederacy, and have wept over its grave; you have acquired character and force of will from the never-failing, but hard-hearted master, experience, and, with a determination worthy of the fair land that gave you birth, it is left for you to fulfill your part in carving from the present chaos a country and a country's rights.

To enable you to grace the high position which your profession will give you, to achieve professional fame, and to reap professional reward, constant labor and a never despairing fortitude must be the controlling principles of your lives. Many dazzling castles will be depicted by a vivid imagination; many will fall in ruins to the ground: block upon block, pillar upon pillar, marble upon marble, you will rear your towering palace to the skies but to behold the magnificent structure come crushing to the earth. Then, my friends, will the heart be tried; then will be generated the stern resolve that reveals the true man or the desolating feeling of despair which destroys the too feeble aspirant. Meet misfortune with a bold front! Learn wisdom from her wintry blasts, and press onward, a wiser though it may be a sadder being. Let her icy hand cool the intellect, but let the heart be preserved in all its freshness and purity! Let not that be chilled! Once touched by the deadening frost, like the sensitive plant, it closes within itself, never to open again till summoned to meet its great Creator. Of late, adversity has dealt severely with us: the meteor-like appearance of our Southern Cross, which, for a time, illumined the horizon of nations and dazzled the world by its brilliancy, has passed from existence, leaving us almost in obscurity; but the youth of the South have only to look to him, who led them with honor, if not success, through the late protracted war, to find an example worthy of imitation. The old warrior, at length overcome by force, is now preparing the coming generation for the intellectual conflict. More honored and respected at the head of the Washington College, than when presiding over the destinies of a new-born nation, the world appreciates the grandeur of the man evincing itself in the philosophy of heroism.

At the commencement of life's career, do not cherish in your bosoms the poisonous thought that you are homeless and countryless, on account of the present unhappy political aspect of the land. It is for you to share in redeeming the fairest portion of the globe from her subordinate position. If our unfortunate South is not to look to her youth for redemption, what hope has she? If they desert her in the hour of her misfortune, she may well submit to the iron heel of despotism without a murmur. The thought is a false one: you have a home; you have a State, and, as a Georgian, I can say, a State and a people upon which the Almighty has impressed his never-failing insignia of greatness. The history of the world establishes the fact that revolutions precede the greatest prosperity; the flow of blood seems to bring forth true virtue, and to add an impetus to the flagging energies of a people. When was Rome arrayed in greater splendor and majesty than in the palmy days of Augustus, whose illustrious reign sprang from the assassination of the noble Cæsar? When has the power of England ever assimilated to that which she exhibited in the time of Cromwell, the regicide? And surely the hero of Austerlitz, whose greatness the mind can scarce compass, was the child of a revolution, and immortalized the age in which he lived by a baptism of fire and blood. Rome, under Augustus,

England, under Charles II., and France, under Louis Napoleon, present illustrations both of the establishment of monarchies from republics and of republics from monarchies—each mutation marked by terrific carnage, destruction and confusion; yet, at what periods have these nations excelled their development under the leaders and forms of government which sprang from revolutions? The ocean never boasts so lofty a crest as when lashed into grandeur and power by the commotion of storms, and the fury of contending winds. Then it is that the rich treasures are heaved up from his agitated bosom to be gathered in the succeeding calm. God grant that we may now be experiencing the last shocks of the huge thunder cloud which has so long overshadowed us; that passion, self consumer, is about to give place to the reign of reason! You and I, my friends, will have long since been gathered to the dust of our ancestors—rivers will have changed their course—the earth have ceased to give forth her fruits—suns to shine and the rains to fall, ere prejudice and fanaticism shall have achieved a final victory over truth and right: the justice of the Almighty is more immutable than the laws of nature.

Let your hearts beat with generous ambition to attain eminence in the profession you have selected—one so elevating to those who appreciate their high calling. Man, the noblest work of God, is your study, and a lifetime of intellectual labor may be expended in acquiring a thorough knowledge of even one minute part of his complicated organization. You are but upon the threshold of the vast science which you must have entirely mastered ere the crown of honor will be awarded you. The discoveries and improvements which expand it are so numerous and rapid that constant application alone will enable you to be familiar with them; and of all pursuits which the varied tastes of man induce him to follow, that of medicine demands, in behalf of mankind, his most thorough comprehension. In embracing it, you assume a heavier responsibility than rests upon your brethren of other professions. Duty to your fellow-man, duty to yourselves, and duty to your God demands that every faculty and every energy which his benign Providence has bestowed, should be exerted, to its utmost extent, to render you efficient in the discharge of the calls that may be made upon you. As a class, you have, in the ordinary course of human events, at least twenty years of intellectual effort before you. What may not be achieved in that time? You must realize that the great mountain of labor is yet to be scaled. You have but rendered yourselves worthy to be called toilers up that rugged step. Schiller has most happily illustrated the necessity of a thorough digestion of all learning to make it available, in a little ballad, termed "Breadth and Depth":

"Many bright wits in the world one sees,
Universal, indeed, in knowledge,
On the charm to attract and the art to please—
Their love could perplex a college.
So fond of the learning they show with such pride,
That she seems, happy men, their monopolized bride.

"And yet they go out of the world quite still,
No trace of existence leaving;
Ah! he who would really the Great fulfill,
And win what is worth achieving,
Must silently gather, and, hour by hour,
In the smallest point, store the amplest power.

"Though the stem may rise proud in the air aloft,
Broad shade through the branches render;
Though the leaves may be bright and their fragrance soft,
'Tis not they that the fruit engender;
From the kernel alone, though so small it be,
Comes the Pride of the Forest: it hides the tree!"

In the discharge of your professional duty you will be carried to the abodes of moral as well as physical anguish. Towering mansions and humble cottages will be the scenes of your labors. "Pallid death beats, with an equal tap at the hovels of the poor, and the palaces of the rich." Your art failing to achieve success, you will be frequent witnesses of the last flashes of life's expiring lamp. You will be often present

"In that dread hour when silent sorrow fears to sigh,
Till all is past"—

some glorious soul, perhaps, having shed its brilliant light upon an admiring world, returning to the great Ruler of the Universe from whom it sprang, once more consigning its earthly tabernacle to kindred sod. The immaterial to the immaterial, the material to the material—such is the inevitable law of nature. It will be for you to sustain suffering humanity in its deepest affliction. The hour of the physician will have given place to that of the true hearted man. On these trying occasions you will be assisted by noble, loving, sympathizing woman.

> "O, woman! in our hours of ease,
> Uncertain, coy, and hard to please,
> And variable as the shade
> By the light quivering aspen made;
> When pain and anguish wring the brow,
> A ministering angel thou!"

O, mothers, wives, sisters, daughters of our sunny South, immortality in this world, and Heaven in the next can but reward you for the untold anguish you have, in days now past, removed from poor, suffering man—inspiriting the hale, cheering the sick, comforting the dying; the departing soul winging "its flight" through "the regions of night," scarce discerning the angel of Heaven before from the angel of earth behind.

Philosophy has taught that no power, once put in motion, is ever entirely lost: the least of you, then, will leave an impress, for good or for evil, upon posterity; and it is your duty to mankind, as well as to yourselves, so to live and act as to furnish worthy examples to coming generations. Though small may be the pebble that is cast into the broad bosom of the deep, the ripples produced by its contact with the surging waters, space can barely measure. The march of civilization is ever onward, and those who do not assist in clearing the path before her are but drones among their fellow-men. They may achieve reputation, may fill for a time the high places of earth, but in the end they must inevitably fall from mischievous distinction to their native obscurity. So let each one of you, my friends, pursue that consciousness of rectitude, implanted by an all-wise Creator in his bosom, that, when summoned to

> "The undiscovered country, from whose bourn
> No traveler returns,"

he may leave a reputation as spotless as the driven snow, a memory enshrined in the hearts of those that knew him—the departing rays of life's setting sun forming a resplendent arch of beauty over his resting place, presenting to the world the soothing and exhilarating reflection, "I THANK MY GOD, I HAVE DONE MY DUTY."

